# Supplementary material for: In Silico Study of the Mechanisms Underlying the Action of the Snake Natriuretic-Like Peptide Lebetin 2 during Cardiac Ischemia
Source: Toxins (Basel). 2022 Nov 11;14(11):787. doi: 10.3390/toxins14110787 (PMC9699598; doi:10.3390/toxins14110787)
Supplement: Supplementary file 1 [file toxins-14-00787-s001.zip › toxins-2008320-supplementary.pdf]

Table S1 Sequence multiple alignments of natriuretic peptides (L2, BNP, ANP, CNP and DNP).

	L2	
**Natriuretic peptide**	**Identity (%)**	**Coverage (%)**
**hANP**	61.90	63.45
**hBNP**	50.00	51.10
**hCNP**	72.73	73.60
**DNP**	45.16	46.47

Data were generated by a homology modeling study using Clustal Omega multiple sequence alignment program server. hANP, human atrial natriuretic peptide; hBNP, human B-type natriuretic peptide; hCNP, human C-type natriuretic peptide; DNP, *dendroaspis* natriuretic peptide; L2, Lebetin 2.

Table S2. Sequence multiple alignments of natriuretic receptors (in human, rat, mouse and chicken).

	NPR-A		NPR-B		NPR-C	
**Natriuretic receptor**	**Identity (%)**	**Coverage (%)**	**Identity (%)**	**Coverage (%)**	**Identity (%)**	**Coverage (%)**
**hNPR *vs* mNPR**	85.41	86.67	98.09	98.7	94.50	95.35
**hNPR *vs* rNPR**	84.71	86.00	97.37	98.00	94.24	95.10
**hNPR *vs* chNPR**	57.35	57.73	69.71	70.61	77.78	78.48
**mNPR *vs* rNPR**	96.47	97.01	99.28	99.00	98.50	99.00
**mNPR *vs* chNPR**	56.16	56.16	69.95	70.85	77.78	78.48
**chNPR *vs* rNPR**	56.87	57.25	70.02	71.00	77.47	78.17

Data were generated by a homology modeling study using Clustal Omega multiple sequence alignment program server. NPR, Natriuretic peptide receptor (NPR); hNPR, human NPR; chNPR, chicken NPR; mNPR, mouse NPR; rNPR, rat NPR.

Table S3. Prediction of 3D models of natriuretic peptides based on DOPE score and total energy.

	L2	BNP	ANP	CNP	DNP
**DOPE score**	-640	-432	-684	-667	-667
**Total Energy** (Kcal/mol)	-441	-634	-757	-166	-734

Data were generated by a homology modeling study using Clustal Omega multiple sequence alignment program server. ANP, human atrial natriuretic peptide; BNP, human B-type natriuretic peptide; CNP, human C-type natriuretic peptide; DNP, *dendroaspis* natriuretic peptide; L2, Lebetin 2.

Table S4. Prediction of 3D models of natriuretic peptides receptors based on DOPE score and total energy.

	hNPR-A	hNPR-B	hNPR-C
**DOPE score**	-54397	-49628	-49108
**Total energy** (Kcal/mol)	-17681	-18384	-17954
	**rNPR-A**	**rNPR-B**	**rNPR-C**
**DOPE score**	-48096	-42863	-43319
**Total energy** (Kcal/mol)	-17780	-18432	-18068
	**mNPR-A**	**mNPR-B**	**mNPR-C**
**DOPE score**	-53649	-40072	-49240
**Total energy** (Kcal/mol)	-18700	-18001	-19111
	**chNPR-A**	**chNPR-B**	**chNPR-C**
**DOPE score**	-45490	-32292	-47418
**Total energy** (Kcal/mol)	-20678	-15802	-19249

Data were generated by a homology modeling study using Clustal Omega multiple sequence alignment program server. NPR, Natriuretic peptide receptor (NPR); hNPR, human NPR; chNPR, chicken NPR; mNPR, mouse NPR; rNPR, rat NPR.

Table S5. Global inter-species comparative study of the binding affinity of natriuretic peptides.

	**Human NPRs**					**Rat NPRs**					**Mouse NPRs**					**Chicken NPRs**				
**NPR-A**	**L2**	**DNP**	**ANP**	**BNP**	**CNP**	**L2**	**ANP**	**DNP**	**BNP**	**CNP**	**ANP**	**DNP**	**BNP**	**L2**	**CNP**	**L2**	**ANP**	**DNP**	**BNP**	**CNP**
ΔG° (kcal.mol)	-25.4*	-25.0	-24.7	-18.0	-17.9	-24.0*	-24.0	-22.9	-16.9	-17.0	-28.2	-25.3	-22.5	-19.9	-18.4	-19.0*	-18.5	-16.6	-15.4	-14.6
SEM	1.0	1.3	0.7	0.4	0.4	0.8	0.2	0.3	0.2	0.2	0.2	0.3	0.2	0.8	0.2	0.2	0.2	0.2	0.2	0.1
**NPR-B**	**DNP**	**CNP**	**L2**	**ANP**	**BNP**	**L2**	**ANP**	**DNP**	**BNP**	**CNP**	**DNP**	**CNP**	**L2**	**ANP**	**BNP**	**L2**	**DNP**	**ANP**	**BNP**	**CNP**
ΔG° (kcal.mol)	-24.1	-22.0	-20.6*†	-19.4	-19.0†	-24.0	-24.0	-22.9	-17.0	-16.9	-28.4	-25.6	-23.9	-23.5	-22.4	-23.1*	-22.1	-19.6	-18.4	-16.7
SEM	0.9	0.8	0.3	0.7	0.8	0.4	0.2	0.4	0.3	0.2	0.4	0.2	0.4	0.3	0.2	0.1	0.4	0.2	0.1	0.1
**NPR-C**	**DNP**	**L2**	**ANP**	**BNP**	**CNP**	**DNP**	**ANP**	**L2**	**CNP**	**BNP**	**ANP**	**L2**	**DNP**	**CNP**	**BNP**	**DNP**	**L2**	**ANP**	**BNP**	**CNP**
ΔG° (kcal.mol)	-25.1	-23.4*	-22.1	-18.3£	-18.2	-16.4	-16.4	-15.5*	-14.6	-14.4	-25.3	-23.0*	-21.6	-19.9	-19.5	-20.5	-20.1*	-17.9	-17.7	-15.7
SEM	0.8	0.7	0.5	0.4	0.4	0.3	0.2	0.2	0.2	0.1	0.2	0.2	0.3	0.2	0.1	0.3	0.2	0.2	0.2	0.2

Data were generated by a molecular docking study using Autodock Vina software. The natriuretic peptides of each line were ranked in decreasing order of affinity to the corresponding natriuretic receptor (NPR-A, -B, -C), i.e. the lower the interaction energy between the receptor and the ligand, the better the stability of the complex. ANP, Atrial natriuretic peptide; BNP, B-type natriuretic peptide; CNP, C-type natriuretic peptide; DNP, *dendroaspis* natriuretic peptide; L2, Lebetin 2. NPR-A, natriuretic peptide receptor A; NPR-B, natriuretic peptide receptor B; NPR-C, natriuretic peptide receptor C. **ΔG°,** Gibbs free energy. n=3 values/condition. *, $p<0.05$ *vs* corresponding BNP; **†**, $p<0.05$ *vs* corresponding NPR-A; £, $p<0.05$ *vs* corresponding NPR-B.

Table S6. Global intra-species comparative study of the binding affinity of natriuretic peptides.

	L2			BNP			ANP			CNP			DNP		
**Human**	**hNPR-A**	**hNPR-C**	**hNPR-B**	**hNPR-B**	**hNPR-A**	**hNPR-C**	**hNPR-A**	**hNPR-C**	**hNPR-B**	**hNPR-B**	**hNPR-C**	**hNPR-A**	**hNPR-A**	**hNPR-C**	**hNPR-B**
ΔG° (kcal.mol)	-24.6	-20.9†	-20.1†	-16.2	-15.8	-14.1	-22.5	-19.9	-17.3	-24.0	-14.5£	-14.2£	-24.4	-22.7	-21.9
SEM	1.0	0.7	0.6	0.4	0.5	0.1	0.8	0.6	0.2	0.3	0.3	0.4	1.5	0.5	0.9
**Rat**	**rNPR-A**	**rNPR-B**	**rNPR-C**	**rNPR-B**	**rNPR-A**	**rNPR-C**	**rNPR-A**	**rNPR-B**	**rNPR-C**	**rNPR-B**	**rNPR-C**	**rNPR-A**	**rNPR-A**	**rNPR-B**	**rNPR-C**
ΔG° (kcal.mol)	-23.3	-17.04†	-17.0†	-16.0	-14.9£	-13.6£	-21.9	-18.8	-17.6†	-19.3	-14.3£	-13.4£	-22.3	-19.0	-18.2
SEM	1.0	0.4	0.3	0.2	0.5	0.0	0.7	0.2	0.4	0.5	0.5	0.3	0.9	0.5	0.4
**Mouse**	**mNPR-B**	**mNPR-C**	**mNPR-A**	**mNPR-A**	**mNPR-B**	**mNPR-C**	**mNPR-A**	**mNPR-C**	**mNPR-B**	**mNPR-B**	**mNPR-C**	**mNPR-A**	**mNPR-A**	**mNPR-B**	**mNPR-C**
ΔG° (kcal.mol)	-18.9	-18.5	-17.9	-18.3	-15.8	-13.5†	-23.8	-19.9	-18.8	-21.5	-14.1£	-13.5£	-22.9	-21.3	-17.6†
SEM	0.4	0.5	0.4	1.0	0.2	0.1	1.2	0.5	0.1	0.2	0.4	0.3	1.0	0.4	0.1
**Chicken**	**chNPR-B**	**chNPR-A**	**chNPR-C**	**chNPR-B**	**chNPR-A**	**chNPR-C**	**chNPR-A**	**chNPR-B**	**chNPR-C**	**chNPR-B**	**chNPR-C**	**chNPR-A**	**chNPR-C**	**chNPR-B**	**chNPR-A**
ΔG° (kcal.mol)	-21.5	-20.1	-19.0£	-15.0	-14.7	-14.4	-18.4	-16.7	-16.5	-17.4	-13.2£	-12.6£	-19.6	-19.2	-17.6
SEM	0.4	0.4	0.4	0.2	0.5	0.4	0.5	0.2	0.3	0.0	0.3	0.2	0.1	0.6	0.6

Data were generated by a molecular docking study using Autodock Vina software. The natriuretic peptide receptors (NPR) of each line were ranked in decreasing order of affinity with the corresponding natriuretic peptide (ANP, CNP, DNP), i.e. the lower the interaction energy between the receptor and the ligand, the better the stability of the complex. ANP, Atrial natriuretic peptide; CNP,C-type natriuretic peptide; DNP, dendroaspis natriuretic peptide; hNPR, human NPR; chNPR, chicken NPR; mNPR, mouse NPR; rNPR, rat NPR. ΔG°, Gibbs free energy. n=3 values/condition. †, $p<0.05$ *vs* corresponding NPR-A; £, $p<0.05$ *vs* corresponding NPR-B.

Table S7. Relative binding energy of natriuretic receptors-L2 and natriuretic receptors-BNP complexes (kcal/mol) using MM/PBSA method.

		hNPR-A	hNPR-B	hNPR-C
**L2**	**BFE** (ΔG°, kcal/mol)	-246.2	-248.0	-232.4*
	SEM	6.2	10,6	9.5
**BNP**	**BFE** (ΔG°, kcal/mol)	-249.3	-255.5	-200.8
	SEM	5.7	8,5	8.8

The free energy of the receptor (NPR)-ligand (NP) was generated by the molecular mechanics/Poisson–Boltzmann surface area (MM/PBSA) binding energy calculations during molecular dynamics simulation. BFE, Binding Free Energy; BNP, B-type natriuretic peptide; L2, Lebetin 2; NPR, Natriuretic peptide receptor (NPR); hNPR-A, human natriuretic peptide receptor A; hNPR-B, human natriuretic peptide receptor B; hNPR-C, human natriuretic peptide receptor C. ΔG°, Gibbs free energy. n=10 values/condition. *, $p<0.05$ *vs* corresponding BNP.

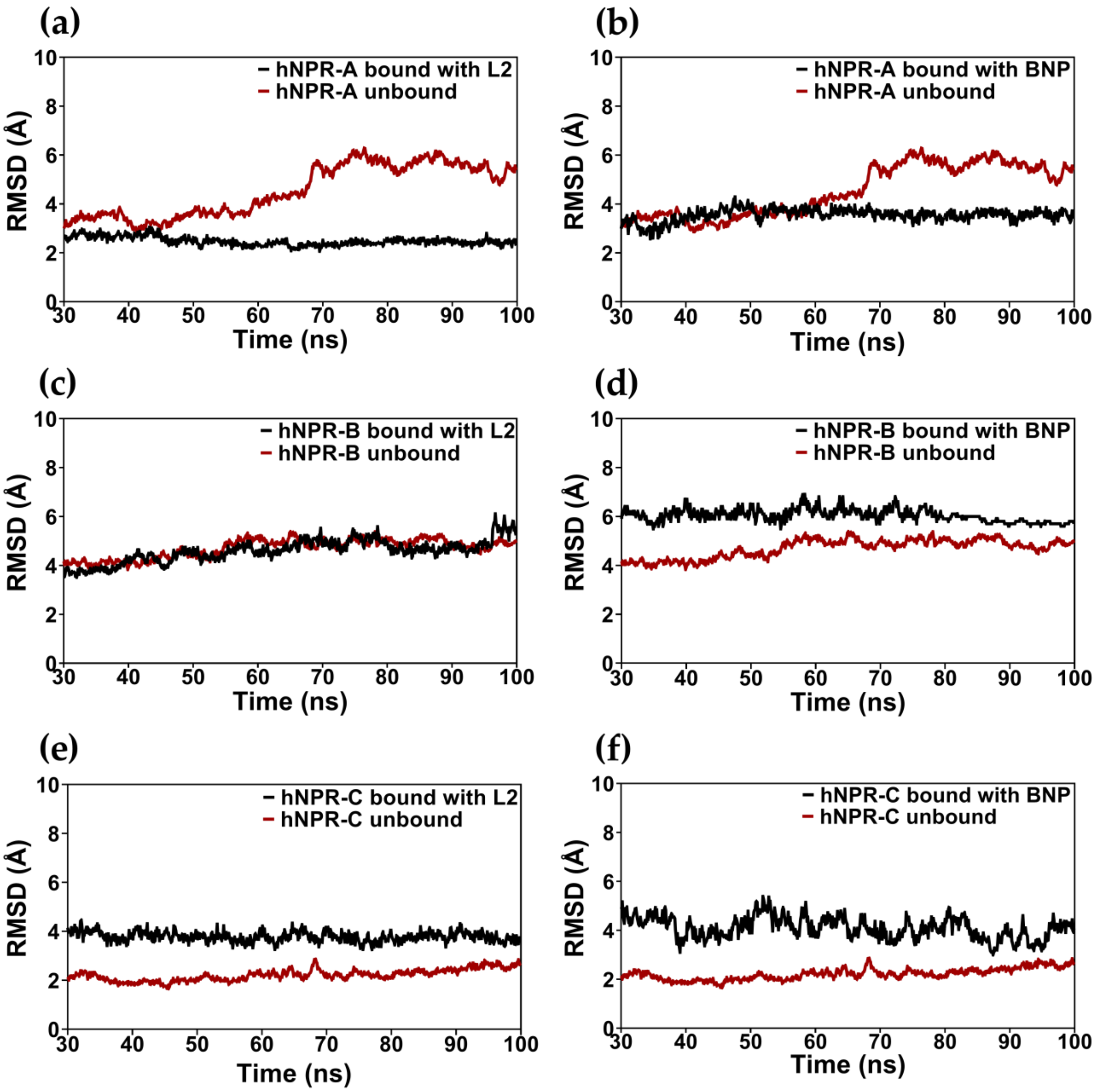

Figure S1. Analysis of root-mean-square deviation (RMSD) plots of human NPRs free receptor (red) and complexed receptor with NPs (black) at 70ns MD simulations. (**a**) RMSD plot of NPR-A unbound and bound with L2. (**b**) RMSD plot of NPR-A unbound and bound with BNP. (**c**) RMSD plot of NPR-B unbound and bound with L2. (**d**) RMSD plot of NPR-B unbound and bound with BNP. (**e**) RMSD plot of NPR-C unbound and bound with L2. (**f**) RMSD plot of NPR-C unbound and bound with BNP.

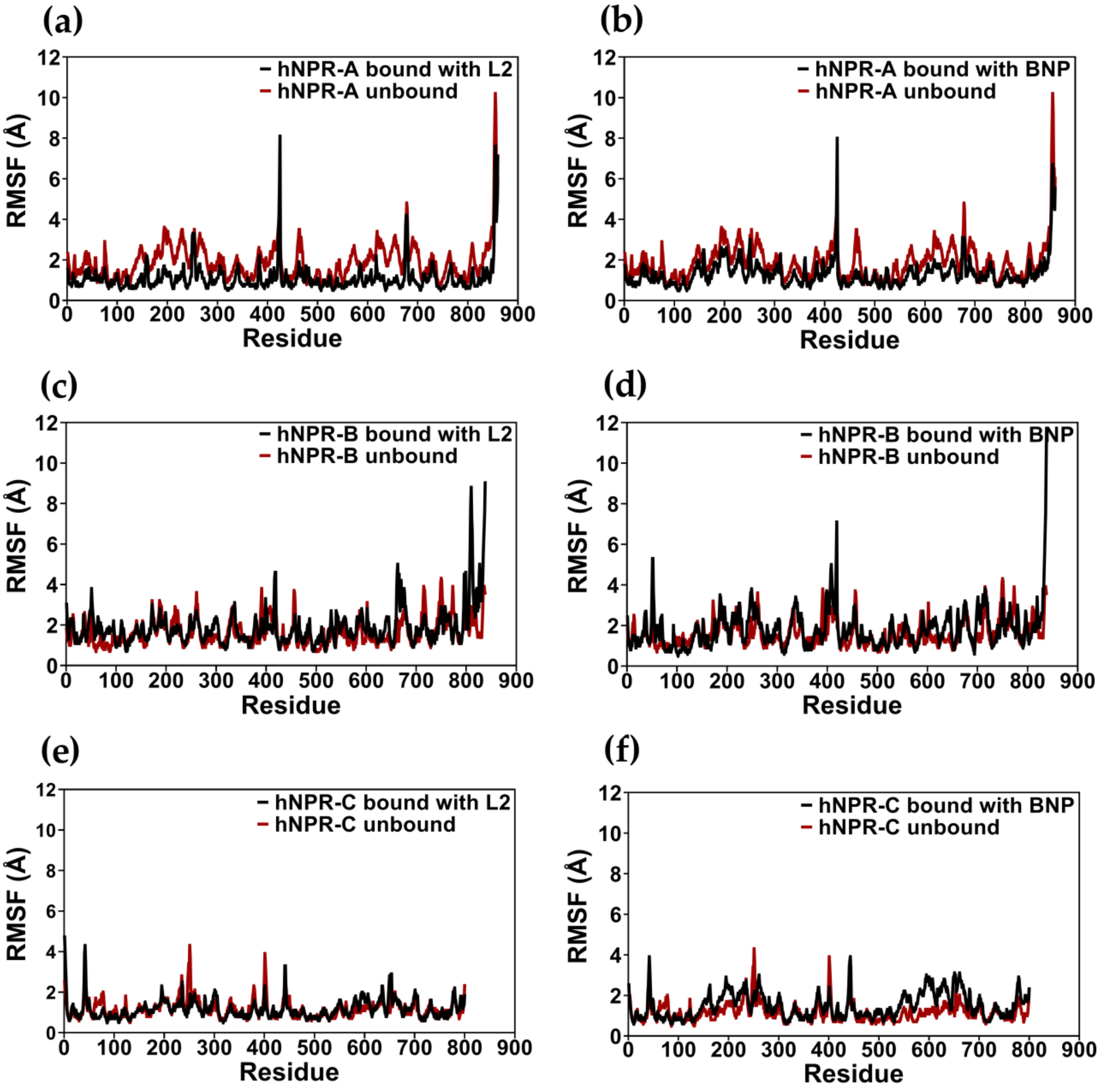

Figure S2. Analysis of root-mean-square fluctuation (RMSF) plots of human NPRs free receptor (red) and complexed receptor with NPs (black) at 70ns MD simulations. (**a**) RMSF plot of NPR-A unbound and bound with L2. (**b**) RMSF plot of NPR-A unbound and bound with BNP. (**c**) RMSF plot of NPR-B unbound and bound with L2. (**d**) RMSF plot of NPR-B unbound and bound with BNP. (**e**) RMSF plot of NPR-C unbound and bound with L2. (**f**) RMSF plot of NPR-C unbound and bound with BNP. RMSF values of the alpha carbon over the entire simulation, where the ordinate is RMSF (Å) and the abscissa is residue.

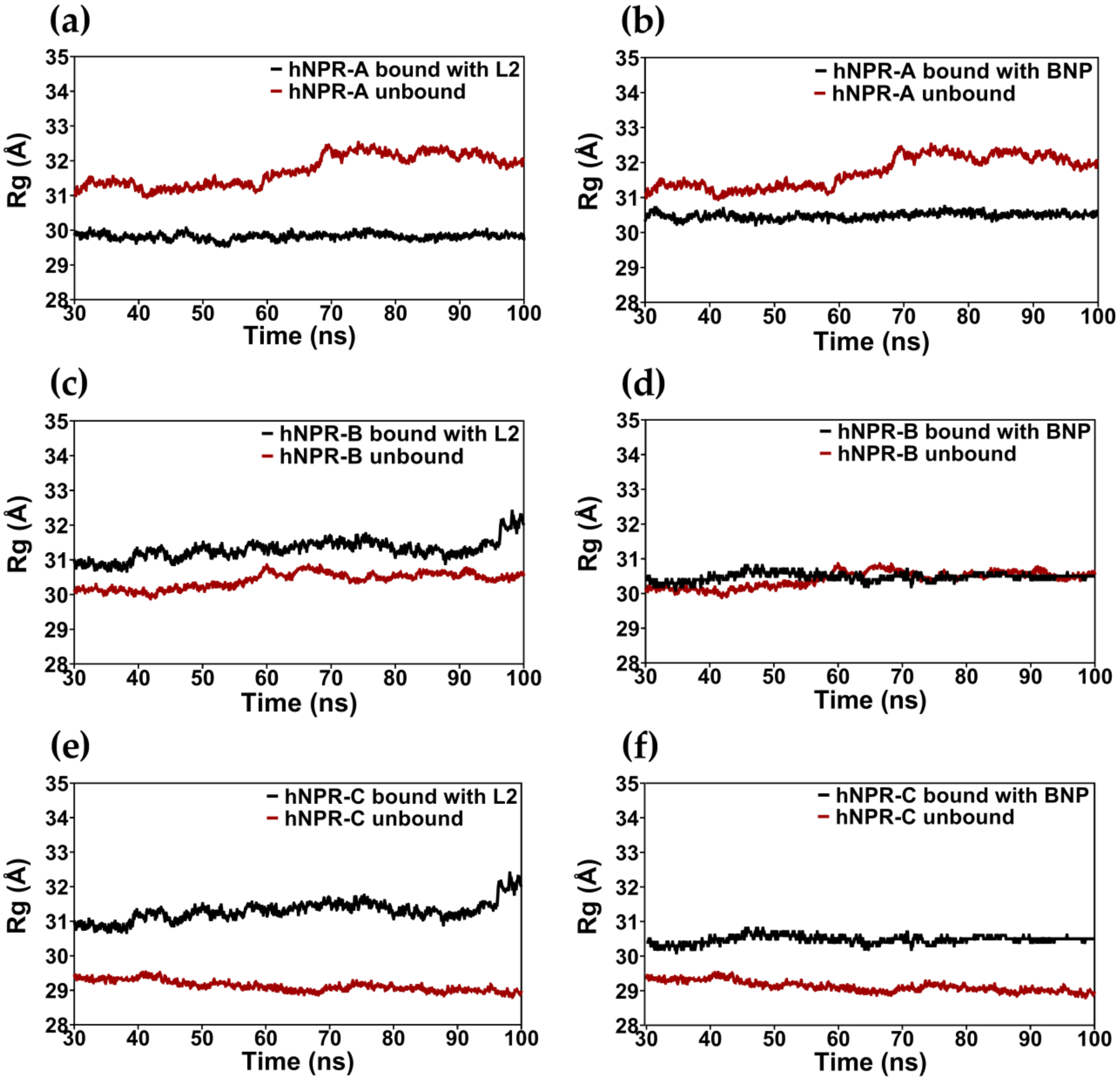

Figure S3. Analysis of radius of gyration (Rg) plots of human NPRs free receptor (red) and complexed receptor with NPs (black) at 70ns MD simulations. (a) Rg plot of NPR-A unbound and bound with L2. (b) Rg plot of NPR-A unbound and bound with BNP. (c) Rg plot of NPR-B unbound and bound with L2. (d) Rg plot of NPR-B unbound and bound with BNP. (e) Rg plot of NPR-C unbound and bound with L2. (f) Rg plot of NPR-C unbound and bound with BNP.

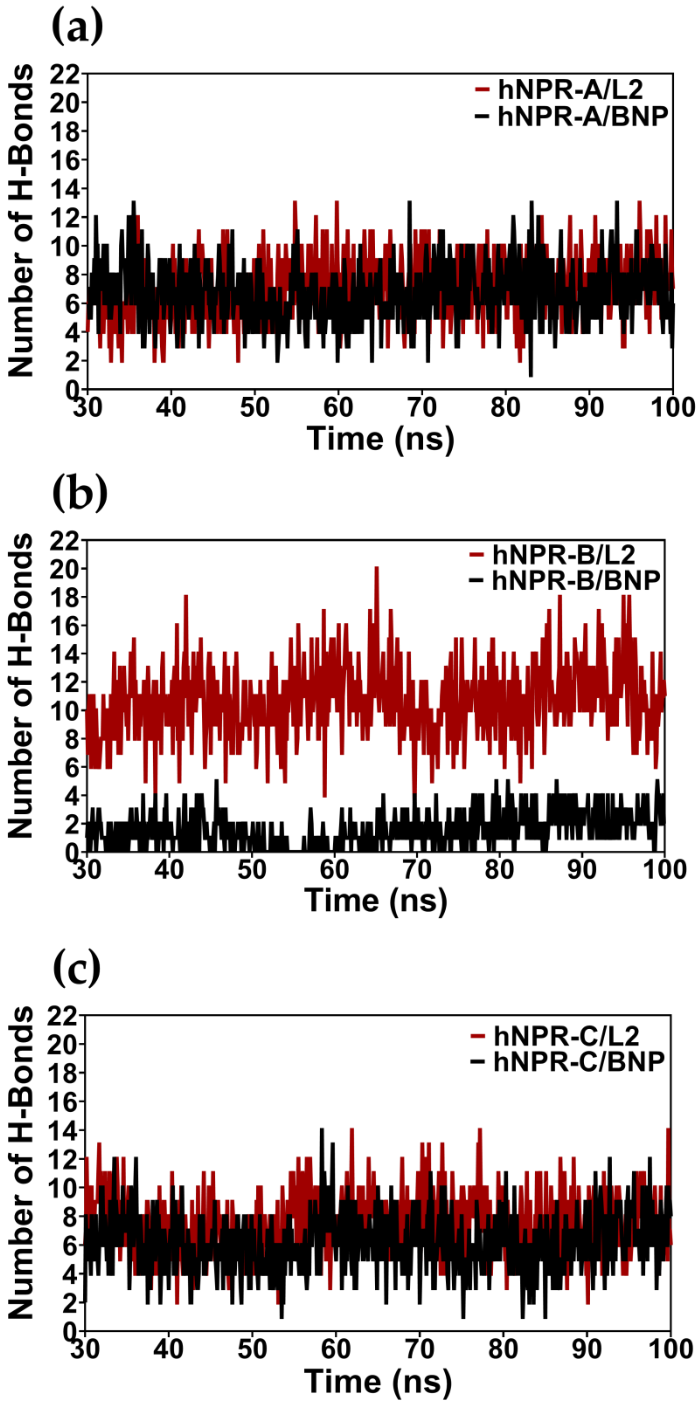

Figure S4. Analysis of the total number of hydrogen (H)-bond throughout the simulation of human NPRs complexed with L2 (red) or BNP (black) at 70ns MD simulations. (a) Difference in the number of H-bond between NPR-A-L2 and NPR-A-BNP complexes. (b) Difference in the number of H-bond between NPR-B-L2 and NPR-B-BNP complexes. (c) Difference in the number of H-bond between NPR-C-L2 and NPR-C-BNP complexes.

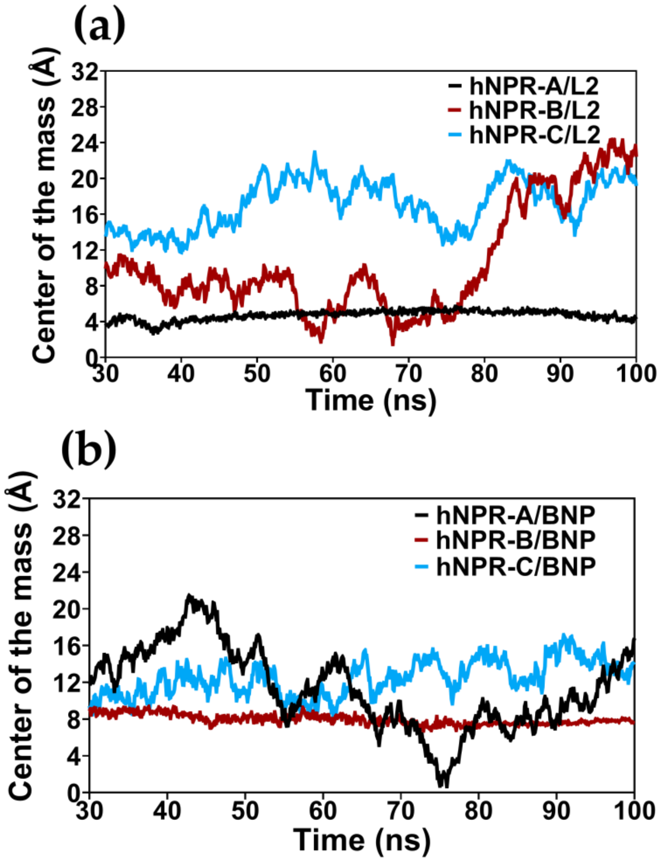

Figure S5. Graphical representation of the trace presenting the distance (Å) between centers of mass of NPR-A (black), NPR-B (red), NPR-C (blue) and L2 (a) and BNP (b) with the variation of the total energy during 70ns.

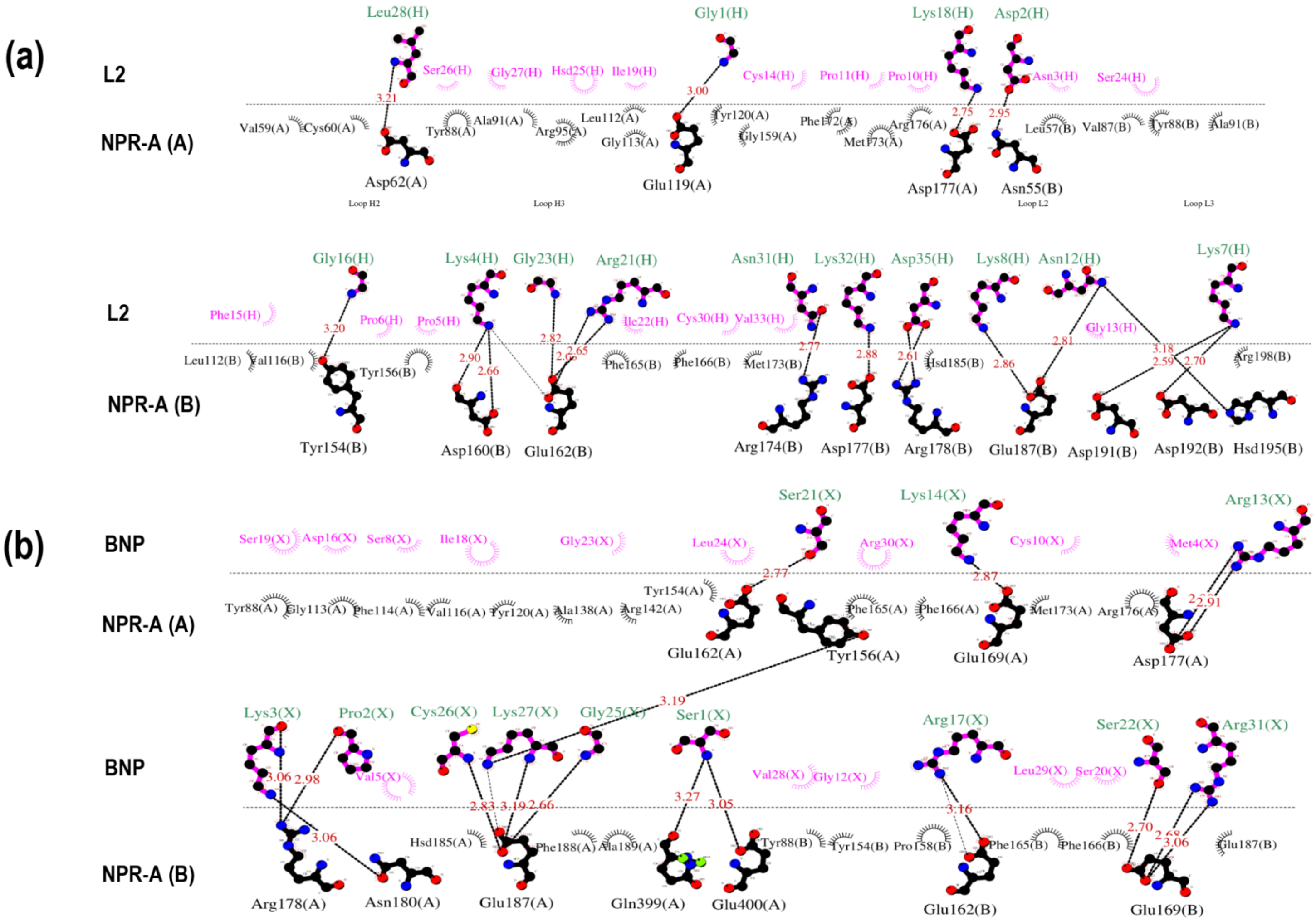

Figure S6. 2D diagrams obtained by LigPlot+ showing the interaction between (a) Lebetin 2 (L2) and (b) B-type natriuretic peptide (BNP) with the active sites of the A and B chains of NPR-A during 100ns of molecular dynamics simulation.

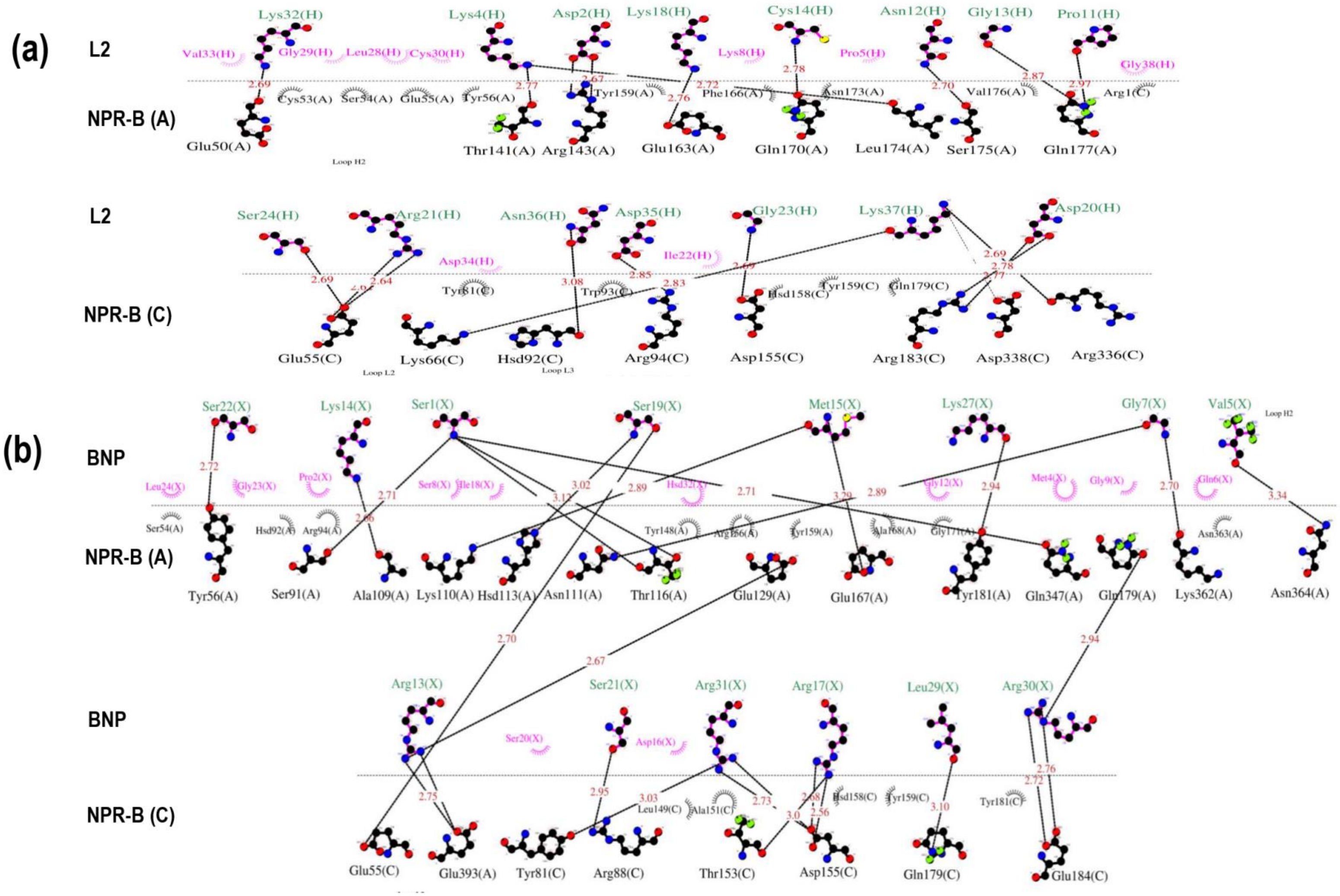

Figure S7. 2D diagrams obtained by LigPlot+ showing the interaction between (a) Lebetin 2 (L2) and (b) B-type natriuretic peptide (BNP) with the active sites of the A and C chains of NPR-B during 100ns of molecular dynamics simulation.

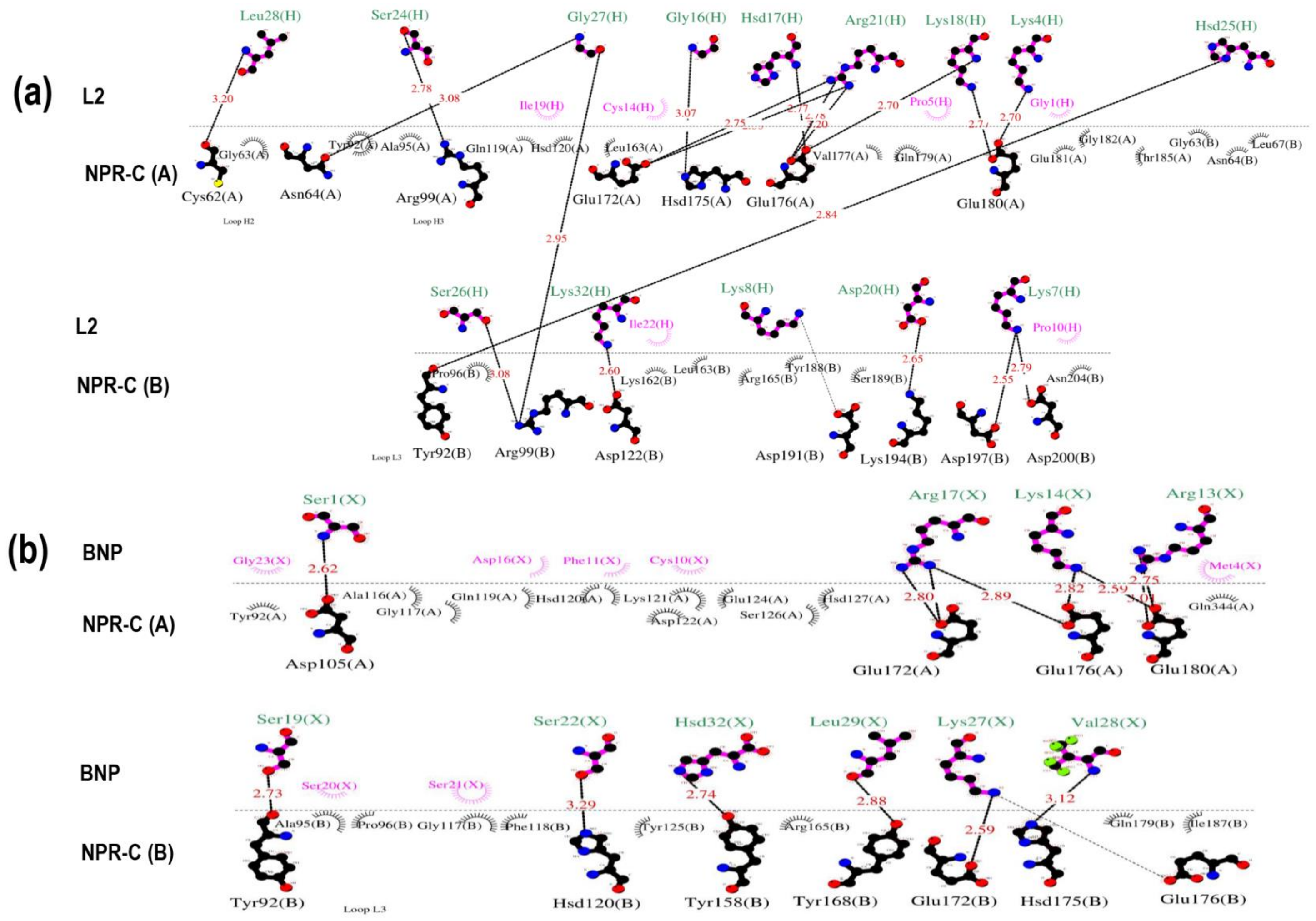

Figure S8. 2D diagrams obtained by LigPlot+ showing the interaction between (a) Lebetin 2 (L2) and (b) B-type natriuretic peptide (BNP) with the active sites of the A and B chains of NPR-C during 100ns of molecular dynamics simulation.
